# Absence of the c.169+50delTAAACAG mutation of *SOD1* gene in a sample of keratoconus patients in Brazilian population

**DOI:** 10.1186/s13104-020-05166-3

**Published:** 2020-07-09

**Authors:** Alessandro Garcia Lopes, Gildásio Castello de Almeida Júnior, Ronan Marques Teixeira, Luiz Carlos de Mattos, Cinara Cássia Brandão de Mattos, Lilian Castiglioni

**Affiliations:** 1 Biology Department, Instituto de Biociências, Letras e Ciências Exatas, IBILCE-UNESP, Universidade Estadual Paulista “Júlio de Mesquita Filho,”, Rua Cristóvão Colombo, 2265, São José do Rio Preto, São Paulo 15054-000 Brazil; 2 Immunogenetics Laboratory, Molecular Biology Department, Faculdade de Medicina de São José do Rio Preto (FAMERP), Avenida Brigadeiro Faria Lima, 5416, Vila São Pedro, São José do Rio Preto, São Paulo 15090-000 Brazil; 3 Ophthalmology Outpatient Clinic, Hospital de Base de São José do Rio Preto, Fundação Faculdade Regional de Medicina (HB-FUNFARME), Avenida Brigadeiro Faria Lima, 5544, São José do Rio Preto, São Paulo 15090-000 Brazil; 4 Epidemiology and Health Department, Faculdade de Medicina de São José do Rio Preto (FAMERP), Avenida Brigadeiro Faria Lima, 5416, Vila São Pedro, São José do Rio Preto, São Paulo 15090-000 Brazil

**Keywords:** Keratoconus, Cornea, *Superoxide Dismutase*-1 gene, Corneal Diseases, Polymorphism, Genetic

## Abstract

**Objective:**

To determine the presence of the 7-bp deletion c.169+50delTAAACAG in intron 2 of *Superoxide Dismutase*-*1* gene in keratoconic patients from the State of São Paulo, Brazil, which promotes splicing variations, resulting in non-functional Superoxide Dismutase-1 antioxidant proteins, which may damage the corneal structure.

**Results:**

A group of 35 keratoconic patients, from whom 35 peripheral blood samples and 58 samples of corneal fragments were evaluated, and a control group of 89 individuals, from whom 41 blood samples and 149 samples of corneal fragments were collected. After the amplification of DNA fragments by polymerase chain reaction, mutational screening analysis was performed by enzymatic digestion, followed by direct sequencing. The absence of the 7-bp c.169+50delTAAACAG mutation in intron 2 of Superoxide Dismutase-1 gene was detected in the analyzed subjects of the 2 groups, both in the cornea and peripheral blood samples. Then, according to our results, there is no involvement of c.169+50delTAAACAG deletion in the pathogenesis of keratoconus in this population, once it was not detected. But we emphasize that studies involving this deletion must be continued in an attempt to elucidate this issue.

## Introduction

Keratoconus (KC) is an asymmetrically bilateral corneal dystrophy characterized by progressive thinning of the cornea, which becomes cone-shaped in a process called ectasia, with a prevalence of approximately 1 in 2000 people [1–7]. It is currently known that KC is a multifactorial disease, involving genetic, environmental, and behavioral factors and etiology remains poorly understood [3, 7–14].

In the literature, several studies define KC as a noninflammatory dystrophy; however, some recently published studies demonstrate an increase in inflammatory mediators (cytokines), such as interleukin-6, in tear samples of affected patients [15–18].

At first, the disease manifests asymptomatically; however, with its progression, visual acuity decreases, culminating in the clinical picture of progressive myopia and irregular astigmatism which may last until the fourth decade of life. Severe cases of KC require corneal transplantation [1, 3, 7, 12, 14, 19–21].

The genetic factors involved, the following are the candidate genes in the etiology of KC: *SOD1, VSX1, LOX, DOCK9,* among others. However, the role of these genes in the disease is still considered complex and controversial [13, 22–25].

Genome-Wide Association Study (GWAS) and other isolated gene association studies have already identified a lot of polymorphisms in more than 60 keratoconus-related loci. But these studies show that the existence of a relevant genetic alteration to the onset of the disease is still unknown, suggesting that further genetic research should be carried out [26–29].

The antioxidant enzyme superoxide dismutase 1 (SOD1) plays a crucial role in corneal physiology; it neutralized superoxide radicals formed owing to exposure to light, which can damage the corneal structure. Interestingly, this enzyme is differently distributed in keratoconic corneas in comparison with that in normal corneas [23, 24, 30, 31].

To date, the most relevant mutation found in *SOD1* and related to KC is a 7-bp deletion, c.169+50delTAAACAG, located in the intron 2 of *SOD1*, which may interfere with the splicing process, generating 2 transcript variants which result in non-functional SOD1 proteins [25, 32, 33].

It is also important to notice that the vast majority of studies on KC have only analyzed blood samples and not cornea, the latter being the target tissue of the disease. Then, to contribute to the genetic knowledge of this important and complex eye anomaly, in this study genetic screening of the intron 2 of *SOD1* was performed to detect the c.169+50delTAAACAG mutation in keratoconic patients from the northwestern region of São Paulo State, Brazil. The search for indexers was found to be unprecedented in the Brazilian population. Another relevant aspect was the analysis of 2 tissues, blood and cornea, in an attempt to detect if the mutation could be acquired.

## Main text

### Experimental population

The participants were divided into 2 groups: the group of keratoconic patients (35 patients: 35 blood samples and 58 corneal samples) and the control group comprising healthy individuals (89 subjects: 41 blood samples and 149 corneal samples) (Table 1), and the corneal fragments and peripheral blood were analyzed in a paired manner.

**Table 1:** Phenotypic characteristics of subjects included in mutational screening of *SOD1* gene

Subjects	Gender	Age (mean/years)	SD	CL(% of individuals)	ER(% of individuals)
KC individuals^a^ (35)	Male (16)	25.1	±6.4	25.7	62.8
	Female (19)	25.2	±5.3		
Control individuals (89)	Male (31)	36.6	±11.1	26.9	22.4
	Female (58)	37.8	±10.7		

^a^KC individuals belonged to isolated cases

SD, standard deviation; CL, contact lens wear; ER, Act of eye rubbing

The samples were collected from the Ophthalmology Outpatient Clinic Hospital de Base (HB-FUNFARME) and of the Visum Eye Center in São José do Rio Preto, SP and were obtained through keratorefractive procedures and corneal crosslinking.

All the patients were subjected to clinical examination and topographic and tomographic evaluations. The clinical check comprised dynamic and static refractions, slit-lamp biomicroscopy, retinoscopy, tonometry, and fundus examination. The diagnosis of KC was based on axial placido disc topography with Easygraph/Topolyzer (Oculus-Wetzlar, Germany), with elevation derived from the anterior corneal curvature map, along with the tomographic examination with Pentacam HR (Oculus-Wetzlar, Germany), based on criteria used in the “Collaborative Longitudinal Evaluation of Keratoconus, CLEK” [34]. In the Pentacam, 3 examinations were performed with optimal fixation and quality, and average values for the following parameters were considered: axial curvature, anterior elevation, posterior elevation, corneal thickness, and BAD III. Elevation data were taken from an 8-mm fixed zone using as reference the Best Fit Sphere manually adjusted.

The following factors were considered as inclusion criteria: absence of previous eye surgery; discontinued use of rigid contact lens (CL) for 4 weeks and gelatinous CL for 2 weeks; absence of previous eye trauma, and absence of primary nasal pterygium. Exclusion criteria were as follows: patients with keratoconjunctivitis sicca, acne rosacea, and severe dysfunction of the meibomian glands; patients using systemic or topical immunosuppressive drugs or those with autoimmune disease; chronic use of eye medication; vulnerable patient owing to physical or mental illness; corneal scar owing to KC; corneal hydrops; infectious keratitis; pregnancy, and lactation.

### DNA extraction, PCR–Random Fragment Length Polymorphism (RFLP) conditions and sequencing

Genomic DNA from peripheral blood (5 ml) was extracted with the QIAamp^®^ DNA Blood QIAGEN kit (Hilden, Germany) according to the manufacturer’s instructions. The genomic DNA from corneal epithelium samples was extracted using the standard phenol–chloroform extraction protocol.

The primer oligonucleotides which were used for PCR amplification of the fragment (approximately 218 bp) comprising part of the exon 2 and part of the intronic region of *SOD1* were as follows: F-CAGAAACTCTCTCCAACTTGC and R-GAGGGGTTTACTGTTTAGGG [22].

PCR reaction mixture (25 µl) containing 100 ng of genomic DNA, 12.5 µl GoTaq^®^ Green Master Promega (WI, USA), 7.5 µl nuclease-free water Promega (WI, USA), and 0.2 pM of each primer (forward and reverse). The cycling conditions comprised an initial denaturation at 95 °C for 3 min, followed by 35 cycles at 95 °C for 30 s, annealing temperature at 59 °C for 30 s, 72 °C for 30 s, and a final extension at 72 °C for 10 min.

RFLP was performed with the restriction enzyme HpyCH_4_ III, which has a restriction site in samples harboring the deletion.

After digestion, the fragments were applied to 2.5% agarose gel and stained with 8 µl Sybr^®^ Safe gel stain (Invitrogen-Thermo Scientific). The electrophoresis lasted 60 min at 100 V in TBE buffer (tris-borate-EDTA), and then the gels were photographed.

Subsequently, samples from the keratoconic group and random samples from the control group were sequenced to confirm the results. The nucleotide sequences were initially analyzed using the Sequence Scanner 2 software (Applied Biosystems) and aligned and edited with the BioEdit 7.0.5 software (Tom Hall, Ibis Therapeutics, Carlsbad, CA).

For reference analyses, the sequences were checked against the National Center for Biotechnology Information GenBank database. The reference sequence used is registered under the accession number NG_008689.1.

## Results

PCR–RFLP of the fragment comprising part of the exon 2 and part of the intronic region of *SOD1* showed no restriction polymorphism in the samples analyzed.

**Fig. 1:**
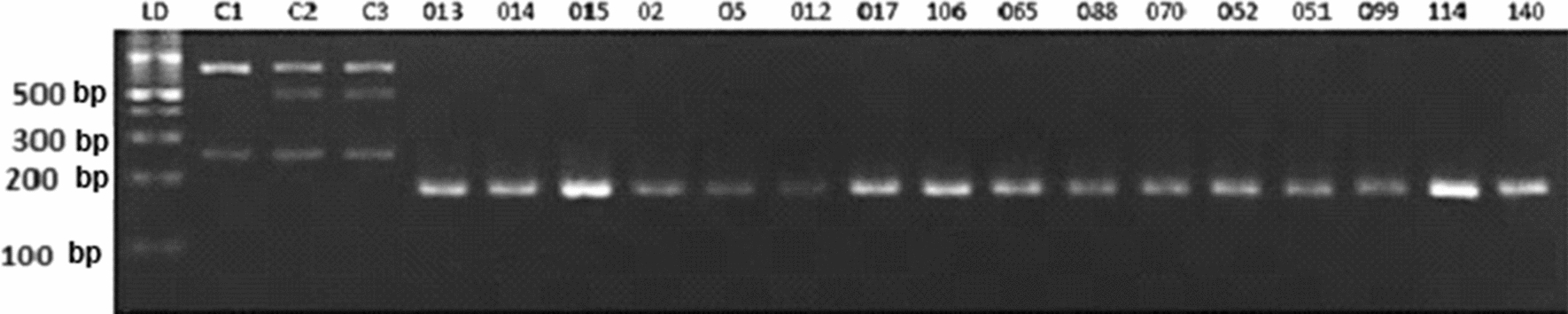
Agarose gel electrophoresis (2.5%) of the fragment comprising part of the intron 2 region and part of the exon 2 region of *SOD1*. The bands indicate fragments of approximately 218 bp that were previously treated with the restriction enzyme HpyCH_4_ III, and which showed no restriction polymorphisms. LD: DNA ladder (100 bp). Keratoconic blood samples (013, 014, 015, 02, 05, 012, 017, and 106) and cornea (065, 088, 070, 052, 051, 099, 114, and 140). C1, C2, and C3: positive reaction controls (ABO blood group gene fragment, which contains the restriction sites for the HpyCH_4_ III enzyme)

Literature data confirm the presence of the c.169+50delTAAACAG deletion in the splicing region and is associated with KC [22, 24, 31, 33]. When present, this mutation promotes alternative splicing and generates 2 mRNA variant molecules, with the loss of exon 2 and/or exons 2 and 3, which results in structural damage and decreased function of the SOD1 protein. None of the samples from this study, either blood or cornea, had a restriction site for the enzyme HpyCH_4_ III, which is capable of cleaving DNA at a specific site promoted by the 7 bp deletion, i.e., the presence of the restriction site would indicate the presence of the deletion, which was not found in our analyses (Fig. 1).

**Fig. 2:**
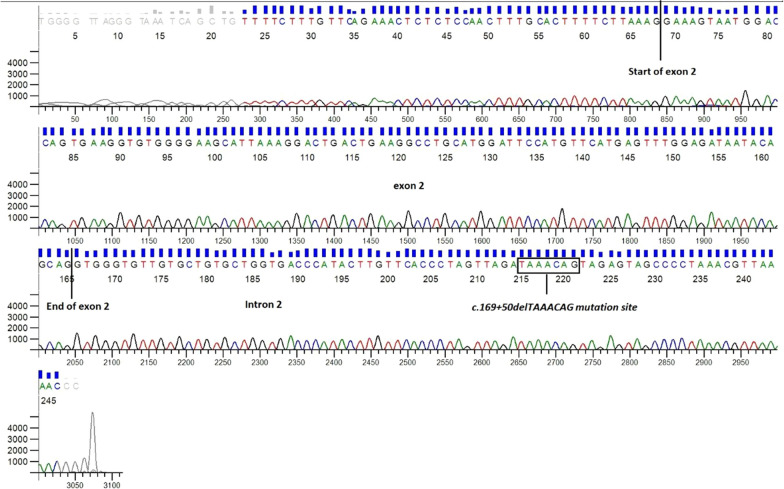
Ectropherogram obtained by direct DNA sequencing of KC samples, showing the absence of the intronic 7-base deletion (c.169+50delTAAACAG) in intron 2 of *SOD1* gene. The data reveal the exon 2 fragment and part of intron 2 in which we can observe the presence of the 7 nucleotides (TAAACAG) corresponding to the mutation site. The sequenced fragments exhibited 100% DNA identity to the *SOD1* gene reference sequence from GenBank database (accession number NG_008689.1)

Direct genotyping and nucleotide sequence analyses of the samples following sequencing did not show any polymorphism and/or mutation in the studied fragment, which is consistent with the results obtained in the gel (Fig. 2).

## Discussion

*SOD1* comprises 9320 bp, encoding a 645-bp transcript comprising 5 exons, resulting in a protein composed of 154 amino acids [33].

Owing to its antioxidant property in corneal physiology, this gene appears to be one of the primary genes related to KC pathogenesis. Several studies of genetic screening found in the literature have investigated this gene as well as other such as *VSX1, LOX, TIMP3, DOCK9,* and collagen genes and identified genetic changes that may be related to the disease. Nevertheless, these results alone are not consistent enough to consider these genetic agents as disease-triggering [23, 24, 35–39].

The genetic variations found in these genes in different populations worldwide are predominantly single nucleotide polymorphisms, which in most cases have a low association with the disease and are considered controversial by several researchers [3, 8, 9, 11, 12, 24, 37, 40].

Specifically, concerning *SOD1*, the most relevant mutation associated with the disease is a intronic 7-bp deletion called c.169+50delTAAACAG, first described in 2006 by Udar et al. [33], through a genetic screening study involving 15 keratoconic patients, of whom 2 presented the mutation. According to the authors, this mutation occurs in the intron 2 of the gene which promotes alternative splicing, resulting in 2 variants of transcripts, which are mRNA molecules with the absence of exon 2 and/or exons 2 and 3, respectively. They generate non-functional SOD1 proteins owing to the loss of their active sites.

De Bonis et al. [22] performed a mutational screening of *SOD1* in 302 Italian keratoconic patients and also identified the presence of the mutation c.169+50delTAAACAG in the intron 2 of the gene in 2 patients. The authors concluded that despite its low frequency (0.9%), this mutation needs to be investigated.

In this study, in a literature review considering some of the most relevant studies on this 7-base deletion published so far [22, 32, 33, 41], we reported a total of 13 KC patients who presented the mutation out in a total of 483 KC patients. Although this study also reported a low frequency of this mutation (2.6%), we emphasize the need for further research on the role of the *SOD1* gene in the KC etiology, due to its great importance in the corneal tissue physiology, as mentioned above.

Moschos et al. [32] also conducted a genetic study involving *SOD1* in a Greek population comprising 33 keratoconus cases and 78 control participants. After DNA sequencing analyses, they identified the presence of 7 bp deletion in 9 patients with sporadic KC. Besides, the mutation appeared in 4 control subjects. Thus, the authors emphasize that the role of this mutation in the KC pathogenesis should be further investigated.

In this literature review, we highlight 2 studies that investigated this deletion in keratoconic individuals from other populations: Al-Muammar et al. [42] and Nejabat et al. [41] investigated 55 Saudi patients and 20 patients from southern Iran, respectively. In contrast to the deletion-positive studies mentioned above, these authors did not detect the mutation.

In the present study involving blood and corneal samples from 35 keratoconic patients, where screening of the intron 2 of *SOD1* was performed, no intronic 7-bp deletion was identified, unlike in other populations. Nevertheless, we emphasize that our result is in agreement with some studies discussed above, which also have reported negative results.

We highlight that this study involving genetic screening of *SOD1* is a pioneer in a Brazilian population. It also stands out for studying rare corneal samples which were collected through surgical procedures.

Finally, we conclude that the genetic factors involved in KC pathogenesis, despite all efforts to date, are poorly defined and controversial. About the presence of the c.169+50delTAAACAG mutation in KC patients, despite the low frequency and weak association with the origin of the disease, it must be considered as, when present, it may trigger the disease or not, depending also on the association of other susceptibility factors, since KC is a multifactorial disease and that environmental and behavioral aspects are involved in its etiology.

## Limitations

- Despite all efforts to date, the genetic factors involved in the pathogenesis of KC are poorly defined and controversial.
- Regarding the presence of the c.169+50delTAAACAG mutation in keratoconic patients, it has an extremely low frequency and when present, may trigger the disease depending on the association with other susceptibility factors because KC is a multifactorial disease and the environmental and behavioral aspects are strongly related to its origin.
- There is no involvement of c.169+50delTAAACAG deletion in the pathogenesis of keratoconus in this population.

## Data Availability

This manuscript is part of the PhD Degree of Alessandro Garcia Lopes, as must be view in UNESP Thesis repository: https://repositorio.unesp.br/bitstream/handle/11449/153175/lopes_ag_dr_sjrp_int.pdf?isAllowed=y&sequence=6. The datasets used and/or analyzed during the current study are available from the corresponding author on reasonable request. The reference sequence of *SOD 1* gene used in this study is registered under the accession number NG_008689.1 in the National Center for Biotechnology Information GenBank database.

## Abbreviations

KC: Keratoconus
CL: Contact lens
*SOD1*: Superoxide Dismutase-1
*VSX1*: Visual System Homeobox 1
*LOX*: Lysyl Oxidase
*DOCK9*: Dedicator of Cytokinesis 9
*TIMP3*: Tissue Inhibitor of Metalloproteinase 3
